# The KLOTHO Birth Cohort: Maternal and Neonatal Vitamin D Status and Neurodevelopmental Outcomes at 10 Years

**DOI:** 10.3390/nu18010076

**Published:** 2025-12-26

**Authors:** Spyridon N. Karras, Dimitrios G. Goulis, Maria Kypraiou, Vikentia Harizopoulou, Antonios Vlastos, Marios Anemoulis, Georgios Tzimagiorgis, Maria Dalamaga, Neoklis Georgopoulos, Evanthia Kassi, Georgios Mastorakos, Kali Makedou, Dimitrios Skoutas, Konstantinos G. Michalakis

**Affiliations:** 1 Laboratory of Biological Chemistry, Medical School, Aristotle University, 55535 Thessaloniki, Greece; tzimagio@auth.gr (G.T.); kalimakedou@gmail.com (K.M.); 2 Unit of Reproductive Endocrinology, 1st Department of Obstetrics and Gynecology, Medical School, Faculty of Health Sciences, Aristotle University of Thessaloniki, 54124 Thessaloniki, Greece; 3 Assisting Nature Centre of Reproduction and Genetics, 57001 Thessaloniki, Greece; mariabioanalysis@yahoo.gr; 4 Medical School, Aristotle University, 54124 Thessaloniki, Greece; vikentiaharizopoulou@hotmail.com; 5 Department of Midwifery, Faculty of Health and Caring Sciences, University of West Attica, 12243 Athens, Greece; antonisvlastos1958@gmail.com (A.V.); mariosanemoulis@hotmail.com (M.A.); 6 Department of Biological Chemistry, National and Kapodistrian University of Athens, 15771 Athens, Greece; madalamaga@med.uoa.gr; 7 Division of Endocrinology, Department of Internal Medicine, School of Health Sciences, University of Patras, 26504 Patras, Greece; neoklisgeorgo@gmail.com; 8 Department of Biological Chemistry, School of Medicine, National and Kapodistrian University of Athens, 10679 Athens, Greece; 9 Second Department of Surgery, Medical School, Aretaieio Athens Hospital, National and Kapodistrian University of Athens, 11528 Athens, Greece; gmastorak@med.uoa.gr; 10 Thermi Clinic, 57001 Thessaloniki, Greece; skoutasd@otenet.gr; 11 Endocrine Practice, Department of Obesity and Metabolism, 11521 Athens, Greece; kostismichalakis@hotmail.com

**Keywords:** vitamin D, 25-hydroxyvitamin D, KLOTHO cohort, pregnancy, neonatal, neurodevelopment, cognition, psychosocial outcomes

## Abstract

Background: Maternal vitamin D status during pregnancy has been hypothesized to influence offspring neurodevelopment; however, the evidence remains inconsistent. Methods: We studied 66 *mother–child pairs* from the KLOTHO cohort with serum 25-hydroxyvitamin D [25(OH)D] measurements at delivery (maternal and umbilical cord). At 10 years of age, neurodevelopment was assessed using standardized questionnaires, generating composite z-scores for cognitive (cognitive, communication, motor) and psychosocial (social–sentimental, special interests) domains. Multivariable models were adjusted for sex, maternal body mass index and education, and neonatal birth weight and gestational age. Results: Maternal 25(OH)D deficiency (<50 nmol/L) was not associated with cognitive composite scores (*p* = 0.77). The psychosocial composite scores showed a non-significant negative trend (*p* = 0.29). Neonatal deficiency showed no consistent association with cognition (*p* = 0.99) or psychosocial outcomes (*p* = 0.30). Exploratory partial correlations suggested a positive association between maternal 25(OH)D and psychosocial development (r = 0.60, *p* = 0.038, n = 12). Seasonal variation in maternal vitamin D was observed (autumn: 56.0 ± 24.6 vs. winter: 32.0 ± 18.3 nmol/L; *p* < 0.0001), but did not translate into differences in 10-year outcomes. Conclusions: In this cohort of 66 pairs, perinatal vitamin D status was not a determinant of global cognition at 10 years of age. A potential link with psychosocial development requires replication in larger longitudinal studies. Due to the limited sample size, all findings should be interpreted as exploratory.

## 1. Introduction

Vitamin D is increasingly recognized as a neuroactive steroid with roles extending beyond calcium homeostasis and bone health. The presence of vitamin D receptors (VDRs) and the enzyme 1α-hydroxylase in the developing brain provides biological plausibility for their role in neurodevelopment, particularly within the hippocampus, prefrontal cortex, and cerebellum, which are regions implicated in cognition, motor control, and socio-emotional regulation [1]. Animal models have demonstrated that vitamin D deficiency during gestation alters brain morphology, neurotransmitter turnover, and synaptic plasticity, supporting the mechanistic basis for long-term neurodevelopmental effects [2]. However, human evidence remains inconsistent. Some longitudinal cohorts have reported that maternal 25-hydroxyvitamin D [25(OH)D] insufficiency is associated with poorer offspring language development, IQ, and behavioral outcomes [3]. In the ALSPAC study, maternal vitamin D concentrations < 50 nmol/L were linked to impaired language and communication skills at 6 years of age [3]. Similarly, data from the Rhea cohort in Greece showed an association between low maternal vitamin D concentrations and adverse neurocognitive outcomes in childhood. In contrast, large prospective cohorts and meta-analyses, have reported null associations between maternal vitamin D status and global measures of cognition or academic achievement.

Beyond cognition, vitamin D has been implicated in psychosocial and behavioral developments. Proposed mechanisms include the modulation of dopaminergic and serotonergic pathways, as well as immunomodulatory effects that could shape emotional and social behaviors [4]. However, the available human evidence is limited, often derived from small sample sizes, and heterogeneous.

While most studies examining prenatal vitamin D exposure have focused on infancy or early childhood, neurodevelopment is a dynamic and prolonged process extending into late childhood and adolescence. Assessment at 10 years of age captures a developmental stage characterized by more stable cognitive, executive, and psychosocial functioning, following periods of rapid neural plasticity in early life. Evaluating outcomes at this time point allows investigation of whether early-life nutritional exposures exert persistent or delayed effects that remain detectable beyond early childhood.

Given these uncertainties, further well-designed prospective studies are required to clarify these issues. The KLOTHO birth cohort, named after the Greek Fate goddess who spins the thread of life, explores the perinatal determinants of child health and development. Within this framework, we aimed to examine whether maternal and neonatal vitamin D status at birth is associated with neurodevelopmental outcomes at 10 years of age, focusing on cognitive and psychosocial domains.

## 2. Methods

Study design and participants. The KLOTHO birth cohort was conducted at the First Department of Obstetrics and Gynecology, Aristotle University of Thessaloniki, Greece, to investigate the perinatal determinants of child health. For the present analysis, we included *66 mother–child pairs* with available maternal and umbilical cord serum samples at delivery and neurodevelopmental follow-up at 10 years of age [5,6]. Recruitment occurred between January 2011 and December 2012. **The inclusion criteria** were full-term singleton pregnancies (37–42 gestational week) with uneventful deliveries. **The exclusion criteria** for mothers were primary hyperparathyroidism, gestational diabetes, morbid obesity (BMI > 30 kg/m^2^), chronic liver or kidney disease, hyperthyroidism, inflammatory bowel disease, rheumatoid arthritis, secondary osteoporosis, and the use of medications influencing calcium or vitamin D metabolism (e.g., corticosteroids, anticonvulsants). The exclusion criteria for neonates were small-for-gestational-age (SGA) status and the presence of major congenital anomalies [5,6]. All mothers provided written informed consent prior to their participation. The study protocol was approved by the Bioethics Committee of the Aristotle University of Thessaloniki (approval no. 1/19-12-2011) and was conducted in accordance with the Declaration of Helsinki.

Maternal and neonatal assessments. Maternal demographic, educational, and social characteristics were recorded at the time of enrolment. Pre-pregnancy body mass index (BMI) was calculated from self-reported weight and height, and the adjusted BMI at delivery was derived by subtracting the expected gestational weight gain. Maternal dietary calcium and vitamin D intake during the third trimester was estimated using a validated semi-quantitative food frequency questionnaire (FFQ) (150 food items) adapted for the Greek population. Maternal alcohol and smoking habits during pregnancy were documented and categorized (none, light, and moderate). Data on ultraviolet B (UVB) radiation (280–320 nm) were obtained from the Laboratory of Atmospheric Physics, Aristotle University of Thessaloniki, Greece. The daily integral of effective UVB radiation (09:00–16:00) during the 45 days preceding blood collection was calculated to represent the ambient exposure.

Biochemical assays. Maternal venous blood was drawn 30–60 min before delivery, and umbilical cord blood was collected immediately after clamping the umbilical vein. Serum samples were stored at –20 °C until analysis. Serum concentrations of 25-hydroxyvitamin D_2_ [25(OH)D_2_] and 25-hydroxyvitamin D_3_ [25(OH)D_3_] were measured using a validated liquid chromatography–tandem mass spectrometry (LC–MS/MS) assay with a lower limit of quantification (LLOQ) of 0.5 ng/mL for each analyte. The assay included analyte purification by liquid–liquid extraction, followed by separation using a chiral microbore column. Accuracy was ensured through external proficiency testing (DEQAS). Total 25(OH)D was defined as the sum of 25(OH)D_2_ and 25(OH)D_3_. Maternal and neonatal vitamin D deficiency was defined as <50 nmol/L (20 ng/mL), insufficiency as 50–75 nmol/L, and sufficiency as 75 nmol/L. Serum calcium (Ca) and phosphorus (P) concentrations were determined using the Cobas INTEGRA clinical chemistry system (Roche Diagnostics, Mannheim, Germany), with intra- and inter-assay CVs < 3.5%. Intact parathyroid hormone (PTH) concentrations were measured using an electrochemiluminescence immunoassay (ECLIA, Roche Diagnostics), with a reference range of 15–65 pg/mL and a functional sensitivity of 6.0 pg/mL.

Neurodevelopmental outcomes. At 10 years of age, children were recontacted, and neurodevelopment was assessed using standardized parental questionnaires (validated for the Greek population). Domains included: cognitive (problem-solving, memory, abstract reasoning), communicational (language comprehension and expression), motor (gross and fine motor coordination), social–sentimental (peer relations, empathy, emotional regulation), and special interests (attention to specific hobbies or activities). Composite z-scores were calculated as follows: Cognitive composite = mean of Cognitive, Communication, and Motor domains and Psychosocial composite = mean of Social–Sentimental and Special Interests domains. Higher z-scores indicate better performance. All neurodevelopmental questionnaires used in this study have been previously validated for use in Greek populations, demonstrating acceptable internal consistency and construct validity. In prior validation studies, reliability indices (Cronbach’s α) for the assessed domains were within acceptable ranges, supporting their use in population-based research settings, acceptable internal consistency and construct validity.

Statistical analysis. Continuous variables were examined for normality using the Shapiro–Wilk test and are reported as mean ± standard deviations (SD) or median (interquartile range). Between-group comparisons (deficient vs. sufficient vitamin D status) were assessed using Student’s *t*-tests, Mann–Whitney U tests, or one-way ANOVA, as appropriate. Categorical variables were compared using the chi-square test. The associations between maternal and neonatal vitamin D status and neurodevelopmental outcomes were evaluated using ordinary least squares (OLS) regression models with heteroskedasticity-consistent standard errors (SE). Effect estimates were expressed as β coefficients with 95% confidence intervals (CI). Exploratory Pearson and Spearman correlations, as well as partial correlations adjusted for covariates, were conducted.

Based on prior evidence, the analyses were adjusted for child sex, maternal pre-pregnancy BMI (kg/m^2^), gestational age at delivery (weeks), birth weight (g), and maternal educational level (elementary, secondary, and higher). Subgroup analyses were performed according to child sex, maternal BMI category (<25, 25–29.9, and ≥30 kg/m^2^), and season of birth (autumn vs. winter). Graphical analyses included residual–residual scatter plots and adjusted estimated marginal means (±SE). Interaction terms were introduced to explore the potential effect of birth weight modification. All analyses were two-tailed, with significance set at *p* < 0.05 and were conducted using SPSS version 23.0 (IBM Corp., Chicago, IL, USA).

## 3. Results

Demographic characteristics of mothers and children are presented in Table 1.

**Figure 1:**
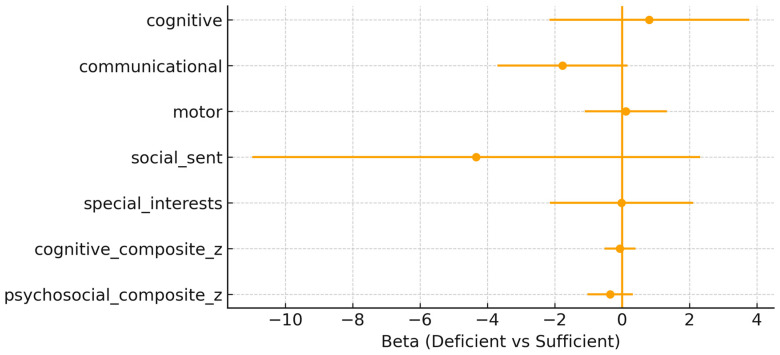
Maternal adjusted β (95% confidence interval) for maternal 25(OH)D deficiency (<50 nmol/L) vs. sufficiency across outcomes.

Maternal 25(OH)D status at the time of delivery. In the fully adjusted models, maternal vitamin D deficiency (<50 nmol/L) was not associated with most neurodevelopmental outcomes at 10 years of age (Figure 1). For the cognitive composite, the effect estimate was β = –0.065 (95% CI: –0.525, 0.395; *p* = 0.774). Similarly, for the psychosocial composite, the effect was β = –0.351 (95% CI: –1.025, 0.324; *p* = 0.294). None of the associations reached significance across the individual domains. A trend toward lower communication scores was observed among children born to vitamin D–deficient mothers (β = –1.770, 95% CI: –3.698, 0.159; *p* = 0.070). No associations were detected for cognitive or motor domains, with wide confidence intervals crossing zero. The wide confidence intervals and standard errors indicate considerable uncertainty and should be interpreted accordingly.

**Figure 2:**
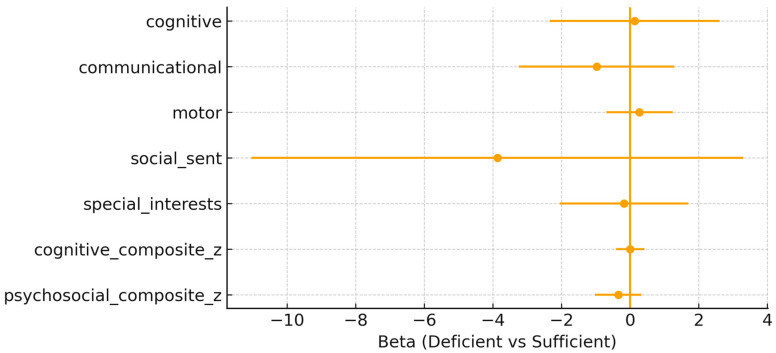
Neonatal adjusted β (95% confidence interval) for neonatal 25(OH)D deficiency (<50 nmol/L) vs. sufficiency across the outcomes.

Neonatal 25(OH)D status at delivery. Similarly, neonatal vitamin D deficiency (<50 nmol/L) showed no consistent association with neurodevelopmental outcomes at 10 years of age (Figure 2). For the cognitive composite, the effect estimate was β = 0.000 (95% CI: –0.415, 0.414; *p* = 0.998), and for the psychosocial composite, β = –0.346 (95% CI: –1.025, 0.332; *p* = 0.303). All associations for individual domains were non-significant, with β coefficients close to zero and confidence intervals broadly overlapping with the null.

**Figure 3:**
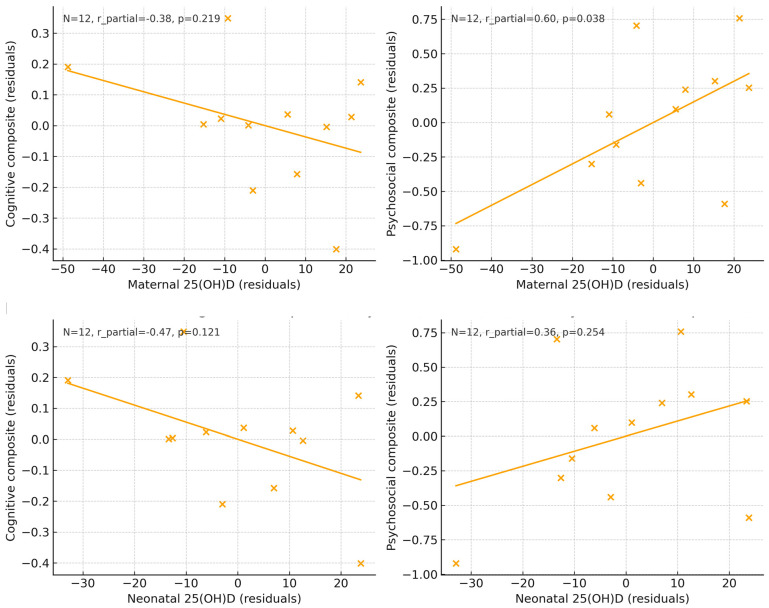
Adjusted scatter plots showing the association between maternal and neonatal 25(OH)D concentrations at delivery and cognitive and psychosocial composite scores at 10 years of age. All variables were residualized for covariates included in the fully adjusted models. Solid lines represent linear regression estimates. Sample sizes for each analysis are displayed within each panel.

**Figure 4:**
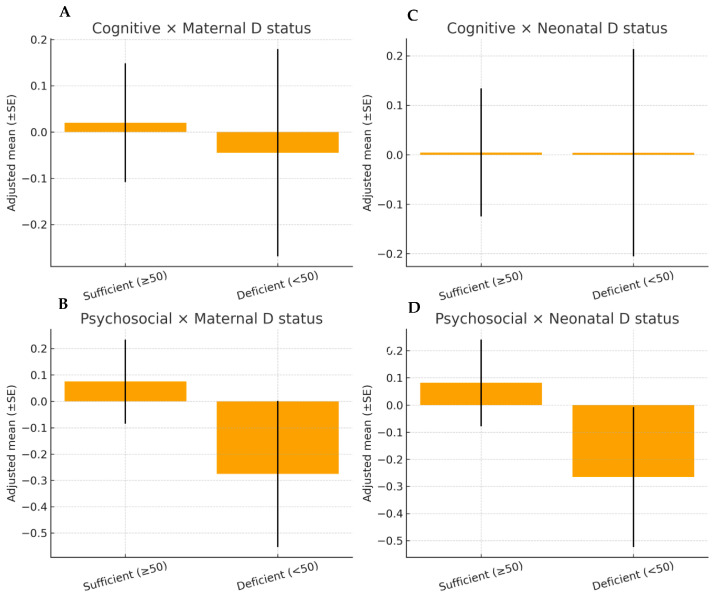
Adjusted estimated marginal means of cognitive and psychosocial composite scores at 10 years of age according to maternal (panels (**A**,**B**) and neonatal (panels (**C**,**D**) vitamin D status at delivery (<50 vs. ≥50 nmol/L). Values represent adjusted means ± standard errors derived from ordinary least squares regression models. Separate panels are shown for maternal and neonatal exposure to facilitate interpretation.

Graphical analyses. The graphical approaches confirmed the regression findings. Adjusted scatter plots of maternal and neonatal 25(OH)D concentrations against cognitive and psychosocial composites (Figure 3) revealed flat regression lines, indicating no associations after covariate adjustment. Likewise, the adjusted estimated marginal means (Figure 4) showed overlapping 95% CI between children exposed to deficient (<50 nmol/L) versus sufficient (≥50 nmol/L) maternal or neonatal vitamin D concentrations.

Subgroup and sensitivity analyses. No significant associations were observed when the analyses were stratified by sex. Among boys, maternal vitamin D deficiency was associated with a higher psychosocial composite score (β = +0.59, 95% CI: –0.03 to 1.21; *p* = 0.061), a trend opposite to our a priori hypothesis. Stratification by maternal pre-pregnancy BMI revealed no consistent association. In the BMI < 25 kg/m^2^ group, maternal and neonatal deficiencies were associated with positive but non-significant coefficients for psychosocial outcomes (maternal β = +0.46, *p* = 0.15; neonatal β = +0.40, *p* = 0.23). No significant effects were observed on cognitive outcomes. Analyses for BMI 25–29.9 kg/m^2^ and ≥30 kg/m^2^ were underpowered due to the small sample size. Interaction models were used to examine whether the association between vitamin D deficiency and neurodevelopment varied by birth weight. For maternal deficiency, a borderline positive interaction with psychosocial outcomes was observed (*p* = 0.060), suggesting that a higher birth weight amplified the positive coefficient. However, this result was modest, inconsistent, and not replicated for cognitive outcomes or neonatal deficiency.

Seasonality of vitamin D concentrations. Maternal and neonatal 25(OH)D concentrations showed strong seasonal variations. Maternal concentrations were significantly higher in autumn (56.0 ± 24.6 nmol/L) than in winter (32.0 ± 18.3 nmol/L; *p* < 0.0001). Similarly, neonatal concentrations were higher in autumn (43.6 ± 18.9 nmol/L) than in winter (22.3 ± 11.2 nmol/L; *p* < 0.0001). Despite this seasonality, stratified analyses did not reveal consistent differences in cognitive or psychosocial outcomes at 10 years.

## 4. Discussion

Prenatal vitamin D is associated with several aspects of brain development. The regulation of Brain-Derived Neurotropic Factor (BDNF) and neurotransmitter regulation has been linked to vitamin concentrations [7]. In this prospective cohort study spanning ten years, we investigated whether maternal and neonatal vitamin D status at birth predicts neurodevelopmental outcomes in late childhood. The principal finding was the absence of consistent associations between maternal or cord blood 25(OH)D concentrations and cognitive or psychosocial composites at 10 years of age. Although isolated trends were observed, such as lower communication scores in offspring of vitamin D-deficient mothers and a borderline positive interaction with birth weight, these effects did not reach statistical significance and should be interpreted with caution.

Our results are exploratory and provide a positive correlation between maternal 25(OH)D concentrations and psychosocial composite scores was observed in a very small subsample; given the limited sample size, this finding should be interpreted with caution.

Our findings are broadly consistent with those of longitudinal studies that have reported null associations between maternal vitamin D concentrations during pregnancy and offspring global cognition or academic performance. Arrhenius et al. found no association between vitamin D and learning deficits, either as a log-transformed continuous variable or as a categorical variable [8]. Sass et al. reported that maternal vitamin D_3_ supplementation during the third trimester of pregnancy did not improve neurodevelopmental outcomes in the first 6 years of the offspring [9]. Brouwer-Brolsma et al. found no association between serum 25(OH)D concentrations and motor fluency or flexibility [10]. In the Generation R cohort, maternal 25(OH)D concentrations during mid-gestation were not associated with nonverbal IQ or language comprehension at 6 years of age [11]. Similarly, a meta-analysis of 12 cohorts found no consistent evidence that maternal vitamin D sufficiency improves overall cognitive development in children. Conversely, earlier studies have suggested protective associations. In the ALSPAC study, maternal vitamin D deficiency (<50 nmol/L) during pregnancy was associated with poorer language and communication skills at 6 years of age [3]. Similarly, data from the Rhea cohort in Greece showed that higher maternal vitamin D concentrations were linked to better cognitive and behavioral outcomes in preschool children. The divergence of results across studies may be related to differences in study design, sample size, timing of exposure assessment, neurodevelopmental instruments used, and residual confounding.

A notable finding of the present study was the marked seasonal variation in maternal and neonatal 25(OH)D concentrations, with significantly higher levels observed in autumn compared with winter. Despite this pronounced seasonal pattern, no corresponding differences in cognitive or psychosocial outcomes at 10 years of age were detected. This dissociation suggests that seasonal fluctuations in late-pregnancy vitamin D status, although biologically meaningful for circulating 25(OH)D concentrations, may not be sufficient to influence long-term neurodevelopmental trajectories when assessed at a single time point. Alternatively, it may indicate that cumulative vitamin D exposure across pregnancy, rather than seasonally driven variation at delivery, is more relevant for neurodevelopmental outcomes. These findings align with evidence from other longitudinal cohorts reporting strong seasonality of vitamin D concentrations without consistent associations with later cognitive or behavioral outcomes. Importantly, the absence of seasonal differences in neurodevelopmental scores should be interpreted in the context of the study’s limited statistical power and the reliance on delivery-only measurements, which may not capture earlier gestational exposure patterns. Nevertheless, this negative result contributes to the broader literature by suggesting that season-related variation in perinatal vitamin D status alone is unlikely to be a major determinant of neurodevelopment at late childhood.

Our neutral findings regarding neonatal 25(OH)D align with reports indicating that cord blood vitamin D may reflect acute maternal status at delivery but does not necessarily predict long-term neurocognitive trajectories. Although our adjusted regression models yielded null findings, exploratory correlations provided suggestive signals. For instance, maternal 25(OH)D was positively correlated with psychosocial composite scores (partial r = 0.60, *p* = 0.038), whereas neonatal concentrations showed modest associations with communication and social–sentimental domains. Although underpowered, these results raise the possibility that vitamin D may exert subtle domain-specific effects rather than broad cognitive effects. This pattern has been highlighted in studies, in which communication and socio-emotional outcomes were more sensitive to prenatal vitamin D status [3].

Although some data relate low vitamin D concentrations with lower visual-motor precision in children, and third-trimester deficiency is linked to poorer executive functioning, these data cover ages only up to 4 years, while our study moves on to late childhood, where some functions seem to be ameliorated. Cantio et al. did report lower full-scale intelligence quotient at the age of 7 years in boys and girls that presented with low cord vitamin D concentrations [12]. Similarly, low vitamin D concentrations in cord blood and early pregnancy are considered risk factors for higher attention-deficit hyperactivity disorder in 5-year-old children, without further observation in later childhood [13].

From a mechanistic standpoint, vitamin D regulates neurotrophic factors, synaptic pruning, and neurotransmitter synthesis [14] and influences immune modulation via cytokines such as IL-6 and TNF-α. These biological actions could preferentially affect socio-emotional development, which is tightly linked to inflammatory signaling and dopaminergic/serotonergic pathways [1,2,4,15]. The absence of significant associations in our cohort may therefore reflect a combination of limited statistical power and the fact that perinatal vitamin D is only one of multiple determinants of long-term neurodevelopment [16,17,18].

Clinically, our findings temper expectations that universal correction of perinatal vitamin D deficiency will translate into measurable gains in childhood cognition [19]. Larger multicenter cohorts, preferably with repeated maternal sampling across gestation and standardized neurocognitive testing, are required to clarify these associations.

Vitamin D has plausible neurodevelopmental roles. Vitamin D receptors are expressed in critical brain regions, such as the hippocampus and prefrontal cortex, and vitamin D modulates neuronal differentiation, synaptic plasticity, and neurotransmitter synthesis [1,2]. Vitamin D also regulates immune signaling, including cytokines implicated in neuroinflammation and emotional regulation [4]. Maternal vitamin D affects brain development during fetal life and causes alterations that may persist into adulthood, leading to the onset of many neurological disorders and diseases [20,21,22,23].

Preclinical studies have indicated that vitamin D regulates inflammatory mediators, protects neurons from apoptosis, and supports neurogenesis and synaptic plasticity [24,25]. With both genomic and non-genomic effects, vitamin D is regarded as a powerful neurosteroid, because the brain can metabolize vitamin D, since neurons and glial cells have CYP24A1 and 1α-hydroxylase [26,27,28]. In addition to the hippocampus, thalamus, hypothalamus, cortex, and substantia nigra, the vitamin D receptor is significantly expressed in many mesencephalic regions during embryonic development, indicating that vitamin D can influence brain maturation and development [29,30,31]. Vitamin D promotes neuronal cell differentiation and death while suppressing proliferation during brain development [32]. However, the modest and inconsistent findings across human cohorts suggest that if such effects exist, they are likely to be subtle and context-dependent, influenced by baseline maternal nutrition, genetic factors, and the early life environment [33,34,35]. The strengths of our study include the use of a well-characterized birth cohort with long-term follow-up, high-quality LC–MS/MS assays for 25(OH)D measurement, and rigorous adjustment for multiple confounding factors. The inclusion of maternal and neonatal samples allowed to disentangle prenatal and perinatal influences.

The choice of assessing neurodevelopmental outcomes at 10 years of age represents both a strength and a conceptual challenge. On one hand, late childhood reflects a stage at which higher-order cognitive and psychosocial functions are more stable and less influenced by transient developmental variability. On the other hand, substantial neurodevelopmental plasticity during infancy and early childhood may partially compensate for early adverse exposures, potentially masking differences that might have been detectable at younger ages. Consequently, the absence of significant associations at 10 years does not preclude the possibility that prenatal vitamin D deficiency may influence earlier neurodevelopmental trajectories. Rather, it suggests that any such effects may attenuate over time or require more sensitive domain-specific assessments to be detected in late childhood. Longitudinal studies with repeated neurodevelopmental assessments across multiple developmental stages are needed to better delineate the timing, persistence, and potential re-emergence of vitamin D–related effects.

However, this study has several limitations. First, the sample size (n = 66 pairs) limited the statistical power to detect modest effects, particularly in the subgroup and interaction analyses and renders the findings exploratory. Second, neurodevelopment was assessed using parental questionnaires rather than direct neuropsychological testing, which may have introduced reporting bias. The reliance on parental questionnaires rather than objective neuropsychological testing may have introduced subjective bias. Third, single time-point vitamin D measurements at delivery may not fully capture gestational exposure, as fetal brain development is sensitive to vitamin D status during mid-pregnancy and may not capture vitamin D exposure during mid-gestation, a critical period for neurogenesis. An important consideration in interpreting our findings relates to the timing of vitamin D exposure assessment during pregnancy. In the present study, maternal and neonatal 25(OH)D concentrations were measured exclusively at delivery, reflecting late gestational status and acute maternal–fetal transfer. However, emerging evidence suggests that vitamin D availability during specific gestational windows may differentially influence neurodevelopmental trajectories. Data from the ECLIPSES study [36] indicate that trimester-specific maternal 25(OH)D concentrations are associated with distinct neurodevelopmental domains, with mid-gestation vitamin D status showing stronger associations with executive function than late-pregnancy measurements.

Similarly, recent work summarized by Wagner and Hollis [37] highlights that fetal brain development is characterized by temporally sensitive periods during which micronutrient availability, including vitamin D, may exert domain-specific effects. Consequently, reliance on delivery-only 25(OH)D measurements may have resulted in exposure misclassification with respect to earlier neurodevelopmentally critical windows, potentially attenuating true associations. This limitation may partly explain the predominantly null findings observed in our analyses, particularly for higher-order cognitive outcomes. Future longitudinal studies incorporating repeated maternal vitamin D assessments across pregnancy are warranted to more accurately delineate the timing-specific effects of prenatal vitamin D exposure on long-term neurodevelopment.

In addition, the observed β values correspond to minimal effect sizes, suggesting limited clinical relevance even where point estimates deviated from zero. Neurodevelopmental outcomes were assessed using standardized parent-reported questionnaires rather than direct neuropsychological testing, which represents an important limitation of the present study. Although these instruments are widely used in epidemiological research and have been validated in Greek populations, parent-reported measures may be influenced by subjective perception, parental expectations, and socio-cultural context. Such reporting bias may reduce sensitivity for detecting subtle or domain-specific neurodevelopmental differences, particularly in older children. Consequently, the absence of significant associations in our study should be interpreted with caution, and future studies incorporating objective neuropsychological assessments alongside parental reports are warranted. Finally, residual confounding from unmeasured lifestyle or genetic factors cannot be excluded from this study.

From a clinical perspective, our results suggest that perinatal vitamin D status may not be a major determinant of long-term global cognition. Given the biological plausibility and inconsistent evidence across cohorts, larger studies with repeated maternal measurements and standardized neurodevelopmental assessments are required. Future studies should also explore gene–nutrient interactions and the role of vitamin D in combination with other micronutrients that are critical for neurodevelopment.

## 5. Conclusions

In summary, the KLOTHO cohort did not demonstrate significant associations between maternal or neonatal vitamin D concentrations at birth and neurodevelopmental outcomes at 10 years. While our findings add to the growing body of evidence suggesting null effects on global cognition, the potential role of vitamin D in specific psychosocial or language domains warrants further exploration. Replication in larger and more diverse populations is essential to clarify the long-term neurodevelopmental relevance of the perinatal vitamin D status.

## Figures and Tables

**Table 1:** Demographic and laboratory features of mothers and neonates. Data are presented as mean ± standard deviation or absolute value (percentage). Abbreviations: BMI: body mass index; PTH: parathyroid hormone; 25(OH)D: 25-hydroxy-vitamin D.

Variable	Value
Mothers’ age (years)	33 ± 6
BMI pre-pregnancy (kg/m^2^)	25 ± 5
BMI term (kg/m^2^)	30 ± 6
Weeks of gestation	39 ± 2
Smoking (%)	None: (73) Light: (17) Moderate: 7 (10)
Education (%)	Primary (19)/Secondary: (60) Higher (21)
Calcium supplementation (%)	(29)
Maternal 25(OH)D (nmol/L)	47 ± 26
Neonatal 25(OH)D (nmol/L)	35 ± 20
Calcium (mg/dL)	9 ± 1
PTH (pg/mL)	27 ± 13

## Data Availability

The original contributions presented in this study are included in the article/Supplementary Materials. Further inquiries can be directed to the corresponding author.

## Supplementary Materials

The following supporting information can be downloaded at: https://www.mdpi.com/article/10.3390/nu18010076/s1, Table S1: Pearson correlations.; Table S2: Spearman correlations; Table S3:Partial correlations (adjusted); Table S4. Maternal and neonatal 25(OH)D concentrations according to the season of birth; Table S5. Sex-stratified OLS (HC3 robust SE): Association of maternal and neonatal vitamin D deficiency (<50 nmol/L) at delivery with 10-year neurodevelopmental outcomes. Table S6. BMI < 25 kg/m^2^ subgroup (OLS with HC3 robust SE): Association between vitamin D deficiency and 10-year outcomes. Table S7. Interaction between birth weight (continuous, g) and vitamin D deficiency at delivery in relation to 10-year outcomes (OLS, HC3 robust SE). Figure S1. Maternal and neonatal 25(OH)D concentrations by season of birth. Maternal and neonatal concentrations were higher in autumn than in winter (Mann–Whitney U test, *p* < 0.0001).
