# Supplementary material for: The KLOTHO Birth Cohort: Maternal and Neonatal Vitamin D Status and Neurodevelopmental Outcomes at 10 Years
Source: Nutrients. 2025 Dec 26;18(1):76. doi: 10.3390/nu18010076 (PMC12787897; doi:10.3390/nu18010076)
Supplement: Supplementary file 1 [file nutrients-18-00076-s001.zip › nutrients-4032591-supplementary.pdf]

Supplementary Material

Table S1. Pearson correlations.

**Exposure**	**Outcome**	**r**	**p**
maternal_25ohd	cognitive	0.215	0.362
maternal_25ohd	communicational	0.463	0.040
maternal_25ohd	motor	-0.195	0.409
maternal_25ohd	social_sent	0.507	0.023
maternal_25ohd	special_interests	0.256	0.277
maternal_25ohd	cognitive_composite_z	0.310	0.183
maternal_25ohd	psychosocial_composite_z	0.488	0.029
neonatal_25ohd	cognitive	0.045	0.851
neonatal_25ohd	communicational	0.481	0.032
neonatal_25ohd	motor	-0.114	0.632
neonatal_25ohd	social_sent	0.461	0.041
neonatal_25ohd	special_interests	0.171	0.470
neonatal_25ohd	cognitive_composite_z	0.259	0.270
neonatal_25ohd	psychosocial_composite_z	0.398	0.082

Table S2. Spearman correlations.

Exposure	Outcome	rho	p
maternal_25ohd	cognitive	0.234	0.320
maternal_25ohd	communicational	0.342	0.140
maternal_25ohd	motor	-0.109	0.647
maternal_25ohd	social_sent	0.518	0.019
maternal_25ohd	special_interests	0.290	0.215
maternal_25ohd	cognitive_composite_z	0.351	0.130
maternal_25ohd	psychosocial_composite_z	0.508	0.022
neonatal_25ohd	cognitive	0.061	0.798
neonatal_25ohd	communicational	0.409	0.074
neonatal_25ohd	motor	-0.029	0.903
neonatal_25ohd	social_sent	0.531	0.016
neonatal_25ohd	special_interests	0.271	0.247
neonatal_25ohd	cognitive_composite_z	0.326	0.160
neonatal_25ohd	psychosocial_composite_z	0.525	0.017

Table S3. Partial correlations (adjusted).

Exposure	Outcome	N	r_partial	p
maternal_25ohd	cognitive_composite_z	12	-0.383	0.219
maternal_25ohd	psychosocial_composite_z	12	0.602	0.038
neonatal_25ohd	cognitive_composite_z	12	-0.472	0.121
neonatal_25ohd	psychosocial_composite_z	12	0.357	0.254

Table S4. Maternal and neonatal 25(OH)D concentrations according to the season of birth.

Group	Season	N	Mean (nmol/L)	SD	Median	Mann–Whitney	p-value
Maternal 25(OH)D	Autumn	37	56.0	24.6	50.5	U = 852.5	<0.0001
	Winter	29	32.0	18.3	25.9		
Neonatal 25(OH)D	Autumn	37	43.6	18.9	41.1	U = 895.5	<0.0001
	Winter	29	22.3	11.2	17.9		

Table S5. Sex-stratified OLS (HC3 robust SE): Association of maternal and neonatal vitamin D deficiency (<50 nmol/L) at delivery with 10-year neurodevelopmental outcomes.

**Sex**	**Exposure**	**Outcome**	**N**	**Beta**	**95% CI (lower, upper)**	**p_HC3**
Boys	Maternal D_def	Cognitive composite	29	-0.12	-0.88, 0.64	0.74
Boys	Maternal D_def	Psychosocial comp.	28	+0.59	-0.03, 1.21	0.061
Boys	Neonatal D_def	Cognitive composite	29	-0.34	-1.01, 0.33	0.30
Boys	Neonatal D_def	Psychosocial comp.	28	+0.47	-0.22, 1.17	0.17
Girls	Maternal D_def	Cognitive composite	35	-0.08	-0.77, 0.61	0.81
Girls	Maternal D_def	Psychosocial comp.	34	+0.25	-0.41, 0.92	0.44
Girls	Neonatal D_def	Cognitive composite	35	-0.09	-0.83, 0.65	0.80
Girls	Neonatal D_def	Psychosocial comp.	34	+0.15	-0.53, 0.84	0.65

Table S6. BMI <25 kg/m^2 subgroup (OLS with HC3 robust SE): Association between vitamin D deficiency and 10-year outcomes.

BMI group	Exposure	Outcome	N	Beta	95% CI (lower, upper)	p_HC3
<25 kg/m^2	Maternal D_def	Cognitive composite	37	-0.11	-0.75, 0.53	0.73
<25 kg/m^2	Maternal D_def	Psychosocial comp.	36	+0.46	-0.17, 1.08	0.15
<25 kg/m^2	Neonatal D_def	Cognitive composite	37	-0.20	-0.83, 0.43	0.52
<25 kg/m^2	Neonatal D_def	Psychosocial comp.	36	+0.40	-0.26, 1.06	0.23

Table S7. Interaction between birth weight (continuous, g) and vitamin D deficiency at delivery in relation to 10-year outcomes (OLS, HC3 robust SE).

**Exposure**	**Outcome**	**N**	**Beta (interaction)**	**95% CI (lower, upper)**	**p_HC3**
Maternal D_def	Cognitive composite	56	+0.00011	-0.00012, 0.00034	0.34
Maternal D_def	Psychosocial comp.	54	+0.00027	-0.00001, 0.00056	0.060
Neonatal D_def	Cognitive composite	56	+0.00005	-0.00018, 0.00028	0.66
Neonatal D_def	Psychosocial comp.	54	+0.00019	-0.00011, 0.00048	0.22

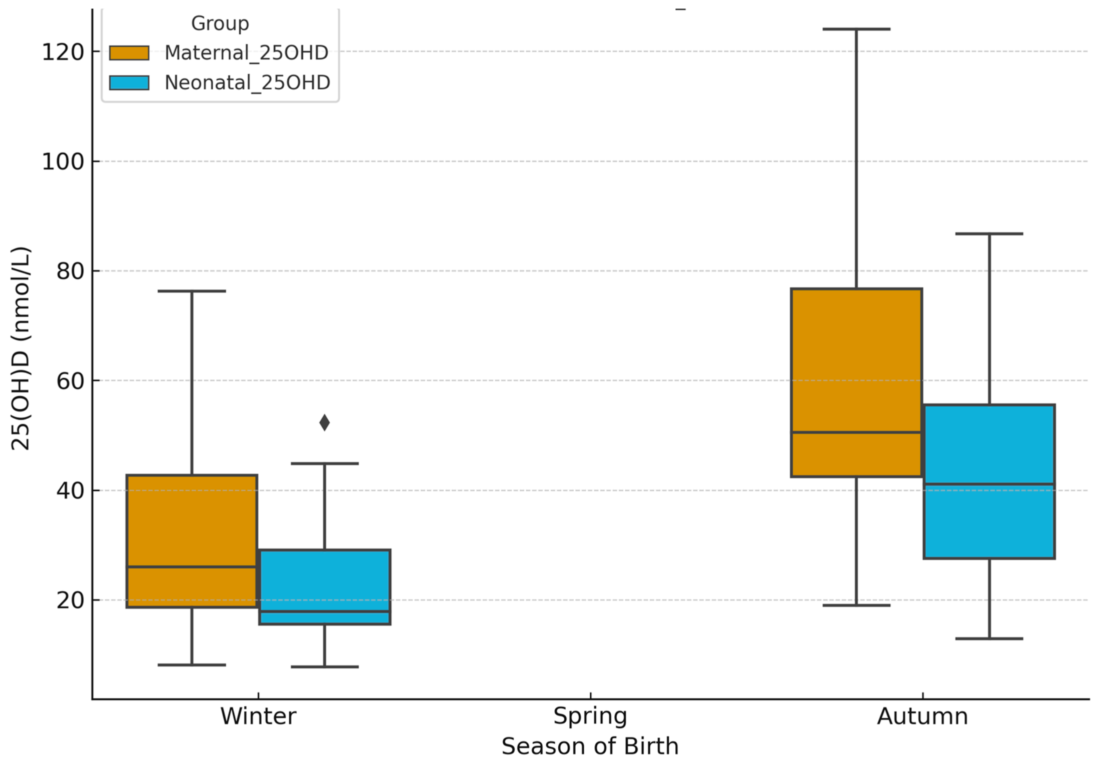

Figure S1. Maternal and neonatal 25(OH)D concentrations by season of birth. Maternal and neonatal concentrations were higher in autumn than in winter (Mann–Whitney U test, $p<0.0001$).
